# Growing season net ecosystem CO_2_ exchange of two desert ecosystems with alkaline soils in Kazakhstan

**DOI:** 10.1002/ece3.910

**Published:** 2013-12-04

**Authors:** Longhui Li, Xi Chen, Christiaan van der Tol, Geping Luo, Zhongbo Su

**Affiliations:** 1 State Key Laboratory of Desert and Oasis Ecology, Xinjiang Institute of Ecology and Geography, Chinese Academy of SciencesUrumqi, China; 2 Faculty of Geo-Information Science and Earth Observation (ITC), University of TwenteEnschede, The Netherlands

**Keywords:** alkaline soil, carbon sequestration, CO_2_ absorption, desert ecosystem.

## Abstract

Central Asia is covered by vast desert ecosystems, and the majority of these ecosystems have alkaline soils. Their contribution to global net ecosystem CO_2_ exchange (NEE) is of significance simply because of their immense spatial extent. Some of the latest research reported considerable abiotic CO_2_ absorption by alkaline soil, but the rate of CO_2_ absorption has been questioned by peer communities. To investigate the issue of carbon cycle in Central Asian desert ecosystems with alkaline soils, we have measured the NEE using eddy covariance (EC) method at two alkaline sites during growing season in Kazakhstan. The diurnal course of mean monthly NEE followed a clear sinusoidal pattern during growing season at both sites. Both sites showed significant net carbon uptake during daytime on sunny days with high photosynthetically active radiation (PAR) but net carbon loss at nighttime and on cloudy and rainy days. NEE has strong dependency on PAR and the response of NEE to precipitation resulted in an initial and significant carbon release to the atmosphere, similar to other ecosystems. These findings indicate that biotic processes dominated the carbon processes, and the contribution of abiotic carbon process to net ecosystem CO_2_ exchange may be trivial in alkaline soil desert ecosystems over Central Asia.

## Introduction

Worldwide paucity of measurements of net ecosystem CO_2_ exchange (NEE) in desert and semi-arid ecosystems limits our understanding on their contributions to global atmospheric carbon cycle (Falge et al. 2002). In the last few years, more and more measurements of NEE have been implemented in some desert and semi-arid ecosystems, including Mojave Desert in the USA (Wohlfahrt et al. 2008), Baja California desert shrub ecosystem in Mexico (Hastings et al. 2005; Bell et al. 2012), Burkina Faso shrub savanna in West Africa (Bruemmer et al. 2008), temperate desert steppe (Yang et al. 2011; Shao et al. 2013), and desert shrub ecosystems (Gao et al. 2012; Liu et al. 2012a,b) in China. The data from these sites indicate that the carbon sequestration capacity by desert and semi-arid ecosystems varies over a wide range. Some papers reported considerably high net carbon uptake by desert ecosystems (Jasoni et al. 2005; Wohlfahrt et al. 2008) and pointed out that desert ecosystem CO_2_ exchange may be more important than previously thought.

Alkaline soils are widely distributed in desert ecosystems, especially around oasis croplands and in areas along dryland rivers where evaporation is quite high but rainfall is low. At the southern periphery of the Gurbantunggut Desert in western China, where oasis agriculture is practiced, alkaline soils were reported to have large ability to sock CO_2_ from atmosphere in an inorganic form, as concluded from a nighttime downward pointed net flux (Xie et al. 2009). Serrano-Ortiz et al. (2010) reviewed abiotic CO_2_ processes in the terrestrial carbon cycle and confirmed that inorganic CO_2_ absorption in alkaline soils can indeed be significant. These findings, combined with other recent papers reporting a high carbon sequestration by desert ecosystems, raise the question whether the long-sought “missing carbon sink” for global carbon cycle can be located in the desert and in semi-arid ecosystems (Stone 2008). However, Schlesinger et al. (2009), by comparing with previous measurements, argued that desert net ecosystem production and carbon pool have been largely overestimated, and that the carbon absorption rates by alkaline soils as reported by Xie et al. (2009) are physically impossible.

Eddy covariance (EC) techniques have commonly been used to measure the NEE between the terrestrial ecosystem and the atmosphere during the past few decades. Although more than 950 site-years of eddy covariance (EC) data have been collected in the international network of FLUXNET (Williams et al. 2009) and the size of EC data is still climbing year by year, data from Central Asian desert ecosystem are still unavailable, resulting in great uncertainties in the carbon sequestration capacity of Central Asian desert ecosystems. Central Asian desert ecosystems account for a large proportion of global dryland area. Due to the high evaporation–precipitation ratio, most of the soils are alkaline with high pH. This offers a good opportunity to investigate the Central Asian desert ecosystem production and employ their contribution to the global land-atmosphere CO_2_ exchange.

The first objective of this study is to quantify the growing season NEE of two desert ecosystems with alkaline soils in Kazakhstan using EC techniques. The second objective is to test the hypothesis that desert ecosystem with alkaline soils acts as a carbon sink at night during growing season. For this purpose, we analyze half-hourly mean NEE data at daytime and nighttime. Finally, this study investigates the responses of NEE to meteorological variables and soil moisture and temperature, in order to interpret the magnitude of maximum uptake ability of CO_2_ absorption by alkaline soil.

## Materials and Methods

### Site descriptions

**Figure 1:**
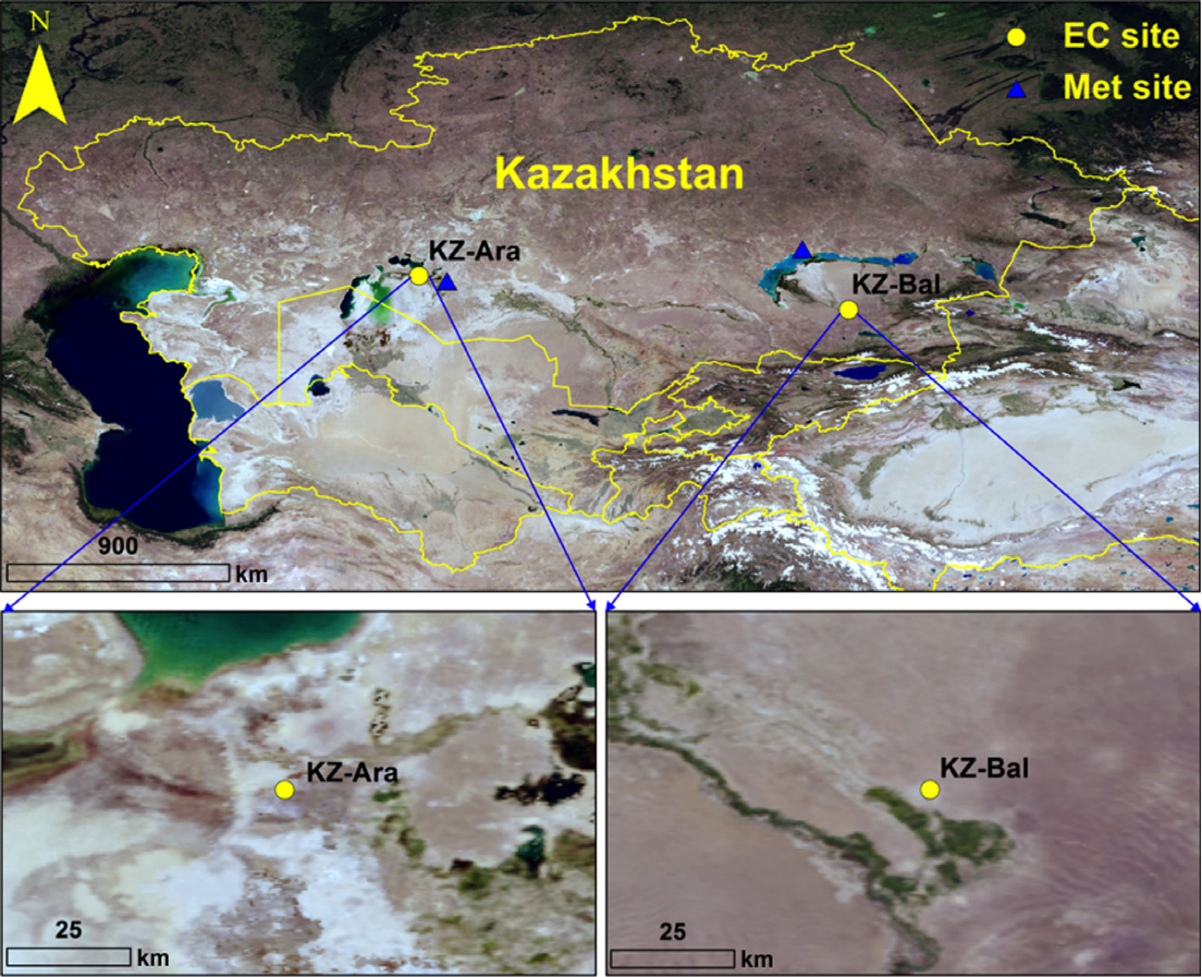
Study area and location of the two eddy covariance (EC) sites in Kazakhstan. Alkaline soil was visually recognized by white pixels in desert regions from satellite image.

We selected two sites in Kazakhstan. One site is close to Aral Sea and the other is close to Balkhash Lake (Fig. 1). Both sites are representative of Central Asian desert ecosystems, and both are dominated by alkaline soils, as one can be visually recognized from satellite images (Fig. 1).

The Aral Sea site (KZ-Ara, 61.08°E, 45.96°N) is located northeast of the Aral Sea. During the past half-century, the surface area of Aral Sea has significantly decreased by 75% (Bai et al. 2011). The KZ-Ara site is actually located at the edge of Aralkum Desert, a man-made desert formed by the desiccated seafloor of the Aral Sea. Presently, the KZ-Ara site is located at 23 km from small Aral Sea coast. Within radius of 5 km of the KZ-Ara site, no residential building or croplands are present. Both vegetation and soil are in various stages of development and temporally and spatially varied. The dominant plant species around the KZ-Ara site are meadow weed grass, reed, and tugaic vegetation (*Elaeagnus oxycarpa*, *Salix* species) in combination with xerophytic dwarf semi-shrub, halophytic shrub, and psammophytic grasses (*Calamagrostis epigeios, Pseudosophora alopecuroides, Phragmites australis, Artemisia terraealbae, Halostachys belangeriana, Tamarix spp., Agropyron fragile*). The fraction of vegetation coverage varied from 0 to 90%, with average value about 30–40% (Breckle et al. 2012). Dominant soil type has been solonchak (medium loamy or loamy) since 1990, and the electric conductivity of soil suspension ranged from 1.33 to 7.38. Soil pH value varied between 8.15 and 8.36 (Breckle et al. 2012). Historical climatic records from the nearest meteorological station Kazalinsk (62.16°E, 45.77°N) with long-term observations showed that annual precipitation is 140.5 mm and mean annual air temperature is 8.3°C (data source: http://ftp://sidads.colorado.edu/pub/DATASETS/NOAA/G02174/).

The Balkhash Lake site (KZ-Bal, 76.63°E, 44.57°N) is located between the Balkhash Lake (200 km away) and the Kapchagay Reservoir (100 km away). The nearest town is Bakbakty, a town along the Ili River, 17 km north of the site. The KZ-Bal site is located at the transect between oasis croplands and original desert habitats. Around 3–5 km west of the site, irrigated crops, well-grown reed, and grasses are distributed. In the south of the site, a small village is resided. Both east and north regions of the KZ-Bal site are original desert ecosystems where desert semi-shrubs, shrubs (*Haloxylon aphyllum, Haloxylon persicum*), and grasses (with relatively large proportion in vegetation component) are distributed. The soil in the KZ-Bal site is takyr-like saline solonchak (Starodubtsev and Truskavetskiy 2011). Historical climatic records from the nearest meteorological station Balkhash (75.08°E, 46.80°N) with long-term observations showed similar amount of annual precipitation (140.2 mm) but lower mean annual air temperature (5.7°C) compared with the KZ-Ara site (data source: http://ftp://sidads.colorado.edu/pub/DATASETS/NOAA/G02174/).

### Eddy covariance and ancillary measurements

In order to investigate the net ecosystem CO_2_ exchange of Central Asian desert ecosystems, two eddy covariance systems have been established to monitor the fluxes of CO_2_, H_2_O, energy, and momentum at KZ-Ara and KZ-Bal in Kazakhstan in April 2012. To measure mean and fluctuating values of vertical, streamwise and lateral wind speed and temperature, a fast response (10 Hz) three-dimensional sonic anemometer thermometer (Wind Master Pro, Gill Instruments, Lymington, UK) was utilized. A fast response (10 Hz) open path gas analyzer (LI-7500, LICOR) was used to measure the mole densities of CO_2_ and H_2_O. Both instruments are mounted at 2.0 m above ground. The dominant wind direction at both sites was on average northeast (Fig. 1), and thus, the head of the sonic anemometer at both sites pointed toward northeast.

Raw data of the three wind components, the speed of sound, and CO_2_ and H_2_O mole densities were acquired and stored at 10 Hz by a data logger (CR5000, Campbell Sci. Inc., Logan, UT). The data are stored in the format of GHG. Each GHG file is an archive containing the raw high-speed data (.data) and information on the study site (.metadata), both in readable text format.

Ancillary meteorological and soil measurements of relevance included photosynthetically active radiation (PAR) flux density (Li-190SA, LI-COR), air temperature and humidity (HMP45C, Campbell), downward and upward shortwave and longwave radiation (CNR-1, Kipp & Zonen, Delft, the Netherlands) at 2.0 m above ground, and precipitation (TE525MM, Texas Electronics, Dallas, TX). Soil temperature (TCAV, Campbell), soil moisture content (CS616, Campbell Sci.), and soil heat flux (HFP01, Hukseflux, Delft, the Netherlands) were measured at 0.20, 0.40, 0.60, 0.80 m depth below the ground. These data were recorded by the data logger at 10 Hz and saved as half-hourly averages.

Up to date, available data covered the period between 30 April and 18 August 2012 at the KZ-Ara site and between 23 May and 6 Sep 2012 at the KZ-Ara site. These data will be used for the analysis in this study.

### Data processing and gap filling

Data processing and gap filling was carried out in three steps. First, GHG files were imported into EddyPro software (version 4.0.0) to calculate out 30 min blocks of flux data. Tilt correction, turbulent fluctuation blocking, time lag compensation, spike detection and removal, and other statistical tests and spectral corrections were applied with the standard functionality of the “Express Model” option in the software. EddyPro also outputs quality flags for all flux variables (sensible and latent heat, momentum, and CO_2_ fluxes) according to Mauder and Foken 2006. During the study period, the average gaps in CO_2_ flux data were 15.7% (836 in 5328) and 30.1% (1544 in 5136) at the KZ-Ara and KZ-Bal sites, respectively.

Second, gap filling was applied in order to derive continuous time series of flux data, required for calculating the daily accumulated CO_2_ flux and the completeness of the data. A Self-Organising Linear Output map (SOLO) artificial neural network (ANN) (Hsu et al. 2002) was employed to fill the gaps in the data flagged with −9999 and 2, resulting from EddyPro software. SOLO “learns” the relationship between 11 input variables (meteorological and soil related) and the interested output flux (CO_2_, latent or sensible heat) using a training data set without any “bad” value. The input data are first classified into five nodes based on Self-Organising Feature Map, so that each node represents an individual region of the input space. At each node, a linear regression is implemented between input variables and the interested output flux variable. Finally, the flux time series with gaps is estimated based on a piecewise linear approximation of the training data set (Hsu et al. 2002).

In EddyPro, spikes were detected as three consecutive outliers, dropping outside a plausibility range defined within a certain time window moving through the time series (Vickers and Mahrt 1997). Detected spikes are replaced by linear interpolation of neighboring values. After this outlier remover, visual inspection showed a small number of spikes remained. Although the number of these data is small, mean and accumulated flux values will be strongly affected. To eliminate this flaw in the data set, the final step of data processing is to implement a Hampel filter for detecting outliers. Outliers detected by Hampel filter with 3 times of variance were replaced with the mean values at the same time in 2 weeks.

## Results

### Climatical and meteorological conditions

**Figure 2:**
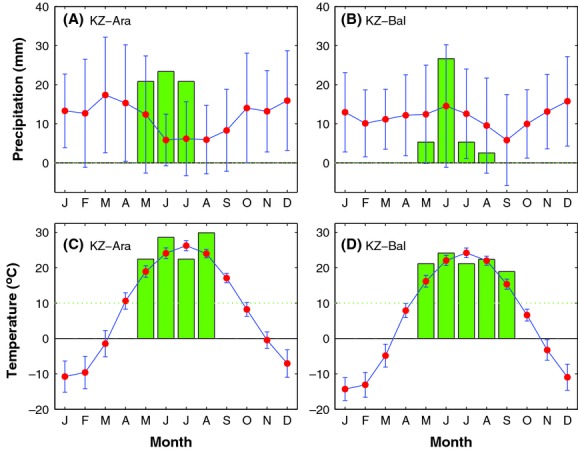
Historical (filled circle with line) and current study period mean monthly (green bars with blue outline) precipitation (A, KZ-Ara; B, KZ-Bal) and mean monthly temperature (C, KZ-Ara; D, KZ-Bal).

Figure 2 shows historically mean monthly precipitation and mean monthly temperature at the KZ-Ara and KZ-Bal sites. Historical annual precipitation at both sites was 140 mm, but mean monthly precipitation exhibited large variability. The KZ-Ara site received lower rainfall in summer season than the KZ-Bal site. At KZ-Bal site, low precipitation occurred in August–October. During the current study period, monthly precipitation was higher than historically mean value at the KZ-Ara site but monthly precipitation in 4 of 5 months was obviously less than historically mean values at the KZ-Bal site (Fig. 2A and B).

Mean monthly temperature at both sites followed a clear sinusoidal pattern in a year. The maximum temperature was 26°C and 24°C in July and the minimum temperature was −10°C and −15°C in January at the KZ-Ara and KZ-Bal, respectively. Mean monthly temperature in 5 months (January–March, November–December) in a year was below 0°C and mean temperature exceeding 10°C was from May to September (Fig. 2C and D). Thus, we defined the period from May to September as growing season. In the current study period, variation of mean monthly temperature basically followed the historical pattern except that mean temperature in July was lower than long-term mean value at both sites.

### Effects of friction velocity on nighttime NEE

**Figure 3:**
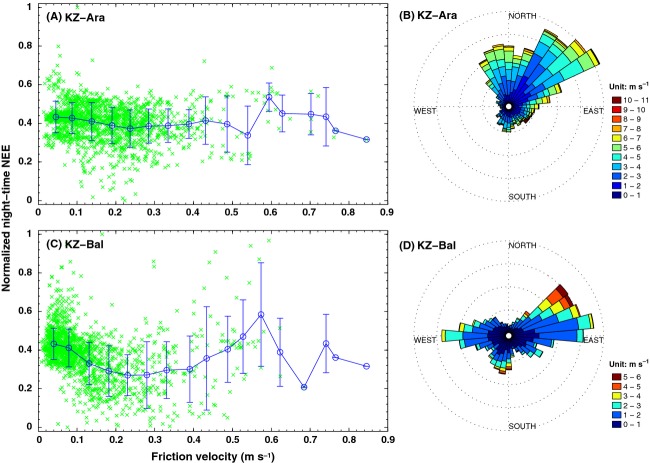
Dependence of normalized nighttime NEE (defined as the ratio of NEE-min [NEE] to max [NEE]-min [NEE]) on friction velocity (left panel), and the wind rose diagram (right panel) at the KZ-Ara and KZ-Bal sites in Kazakhstan. Symbol with “x” represents half-hourly data during the study period, and open circle indicates bin-averages of 0.05 m s^−1^ 519 width. Error bars refer to ±1 standard deviation.

EC-measured nighttime NEE in low turbulence conditions may be subject to systematic bias, and the dependence of nighttime NEE on friction velocity (u*) could vary site by site (Anthoni et al. 2004). Relating nighttime NEE and u* helps to identify the uncertainty caused by low turbulence. At the KZ-Ara site, nighttime NEE (i.e., ecosystem respiration) was independent of u* in a broad range between 0 and 0.9 m s^−1^ as shown by a relatively horizontally linear regression between normalized nighttime NEE and u* (Fig. 3A). A wind rose diagram showed that the dominant wind flow direction is northeast (Fig. 3B) where vast desert region was (Fig. 1). At the KZ-Bal site, the nighttime respiration was influenced by u*, especially under very low turbulence conditions (u*<0.15 m s^−1^) (Fig. 3C). The dependence of nighttime NEE on u* may be partly explained by the heterogeneous landscapes around the site (see *Site descriptions*) and wind direction distribution (Fig. 3D). At the KZ-Bal site, considerable wind flows were from the west (Fig. 3D) where oasis croplands were distributed (Fig. 1), and hence, EC measurement may be impacted. In contrast, dominant wind directions were northeast and north, where only desert shrub communities were present (Fig. 1) at the KZ-Ara site. However, the NEE data under low u* conditions were flagged as “bad” values and replaced by SOLO estimations. Further, the difference between the maximum and minimum normalized nighttime NEE was only 38% of the average nighttime NEE; thus, the impact of the development of turbulence (u*) on nighttime respiration is apparently small (Fig. 3C).

### Diurnal variations of NEE

**Figure 4:**
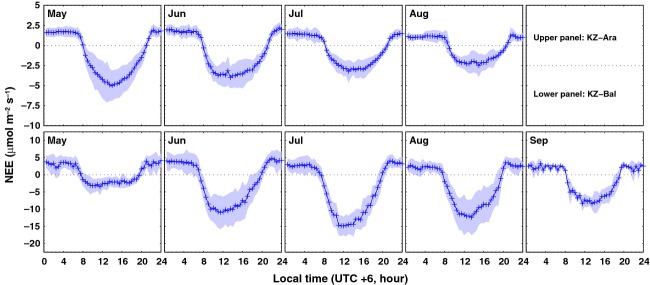
Diurnal courses of mean NEE during study months from May to September at the KZ-Ara (upper panel) and KZ-Bal (lower panel) sites. Shaded areas represent ±1 standard deviation.

The mean diurnal NEE in each month followed a clear sinusoidal dynamic during the growing season (Fig. 4). Mean diurnal variations of NEE at each month showed a net carbon uptake (negative NEE) at daytime and a net carbon release (positive NEE) at nighttime at both sites. The peak NEE occurred at 12:00 local time for all months at both sites (corresponding to a local solar time of 10:00 for KZ-Ara and 11:00 for KZ-BAL). Diurnal maximum rates of carbon uptake varied per month, and the highest amplitudes during the study period were observed in May and July, while mean uptake rates reached up to −5.0 and −15.0 *μ*mol m^−2^ s^−1^ at the KZ-Ara and KZ-Bal sites, respectively. The significant difference in maximum carbon uptake rates between the two sites and the difference in the month of peak carbon uptake rates were possibly related to the vegetation compositions and climatic conditions. At the KZ-Ara site, the vegetation around the site was all desert plants, shrubs, or grasses and no human disturbance applied. The temperature at the KZ-Ara site is higher than that at the KZ-Bal site, which may cause earlier phenology for plants' primary production. At the KZ-Bal site, the observed NEE may be impacted by surrounding oasis crops and reed and grasses grown alongside the acequia.

**Figure 5:**
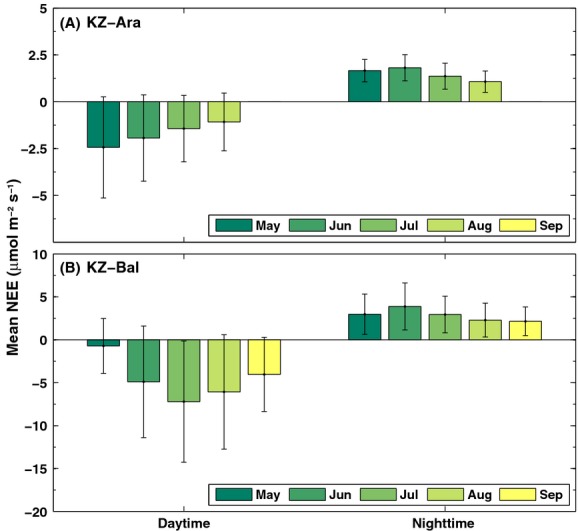
Mean monthly NEE at daytime and nighttime at the KZ-Ara (A) and KZ-Bal (B) sites. Error bars represent ±1 standard deviation.

Both sites show a typical pattern of net carbon uptake at daytime and net carbon release at nighttime (Fig. 5). At the KZ-Ara site, the daytime mean monthly NEE ranged from −2.5 *μ*mol m^−2^ s^−1^ in May to −1.1 *μ*mol m^−2^ s^−1^ in August. The daytime mean NEE decreased from May to August. In contrast, mean monthly nighttime NEE ranged from 1.65 *μ*mol m^−2^ s^−1^ in May and June to 1.0 *μ*mol m^−2^ s^−1^ in August (Fig. 5A). At the KZ-Bal site, daytime carbon uptake rates during May–September months ranged from −0.7 *μ*mol m^−2^ s^−1^ in May to −7.2 *μ*mol m^−2^ s^−1^ in July. The differences among months were obvious, and the peak carbon uptake rate was in July. The mean nighttime ecosystem respiration ranged between 2.15 *μ*mol m^−2^ s^−1^ in September and 3.88 *μ*mol m^−2^ s^−1^ in June (Fig. 5B). At both sites, the maximum mean nighttime ecosystem respiration occurred in June, different from the months of maximum mean daytime NEE.

### Dependency of daytime NEE on PAR

**Figure 6:**
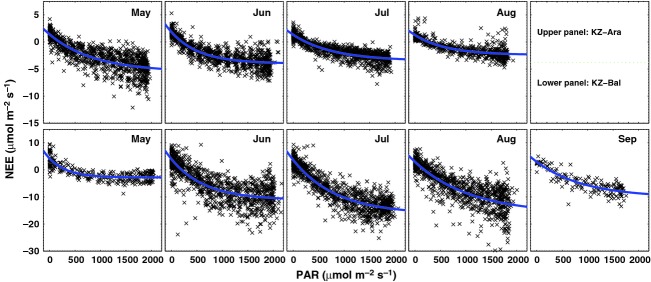
Dependency of half-hourly mean NEE on photosynthetically active radiation (PAR) during the study period (May–September) at the KZ-Ara (upper panel) and KZ-Bal (lower panel) sites. The blue curve was fitted with a first-order exponential decay model in the form of NEE (µmol m^−2^ s^−1^) = *A*exp(−PAR/*B*) + *C*. All model parameters were listed in Table 1.

**Figure 7:**
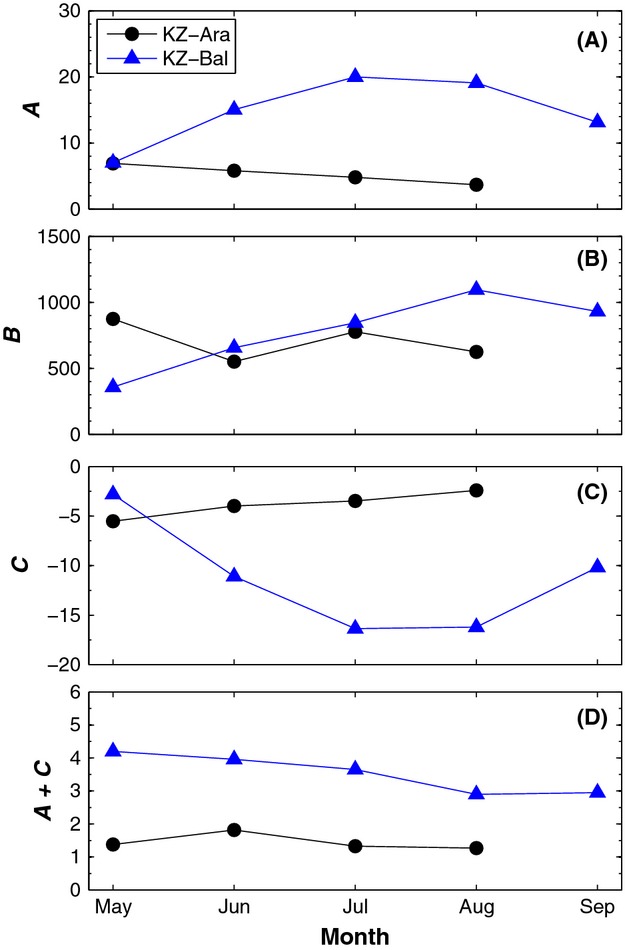
Monthly variations of parameter values in the fitted first-order exponential decay model (NEE = *A*exp(−PAR/*B*) + *C*) between daytime NEE and PAR at the KZ-Ara (black circle) and KZ-Bal (blue triangle) sites. All model parameters were listed in Table 1.

The dependence of daytime NEE on PAR was assessed by fitting a first-order exponential decay model in the form of NEE (*μ*mol m^−2^ s^−1^) = *A*exp(−PAR/*B*) + *C* (Fig. 6) for every month. All parameters and the square of the correlation coefficient are listed in Table 1. The parameter *C* in the fitting equation represented the CO_2_ uptake saturation threshold: Its absolute value represents the maximum uptake that occurs if photosynthesis is light saturated. The sum *A* + *C* (where *C* has a negative and *A* a positive value) represents nighttime respiration. Monthly variations of parameter *C* indicate seasonal changes in maximum CO_2_ uptake. A comparatively good match between the measured data and the resulted model output was derived for the months with the highest net CO_2_ exchange (parameter *C* in the fitting equation), that is, May (*C* = −5.53 *μ*mol m^−2^ s^−1^, *R*^2^ = 0.66) at the KZ-Ara site and Jul (*C* = −16.36 *μ*mol m^−2^ s^−1^, *R*^2^ = 0.82) at the KZ-Bal site (Table 1 and Fig. 7A). Similar to the absolute value of parameter *C*, the value of *A*+*C* is a factor three higher at the KZ-Bal site than at the KZ-Ara site (Table 1 and Fig. 7D). The seasonal cycle exhibited by *C* is not present in *A* + *C*: nighttime respiration appears to be much more constant than daytime maximum (absolute) NEE (Fig. 7D).

**Table 1:** Parameters of the exponential decay model in Figure 7. The model was expressed as NEE (*μ*mol m^−2^ s^−1^) = *A*exp(−PAR/*B*) + *C*, where PAR is photosynthetically active radiation (*μ*mol m^−2^ s^−1^), and *A*, *B*, and *C* are fitting parameters. *R*^2^ is square of correlation coefficient between the measured and modeled NEE. *C* represents the maximum uptake, while *A+C* represents nighttime respiration

	KZ-Ara				KZ-Bal				

	May	June	July	August	May	June	July	August	September
*A*	6.91	5.81	4.81	3.68	7.01	15.05	20.01	19.1	13.13
*B*	874.48	551.12	777.08	624.87	358.33	656.46	845.4	1095.4	930.59
*C*	−5.53	−3.99	−3.48	−2.41	−2.81	−11.09	−16.36	−16.2	−10.18
*A + C*	1.38	1.82	1.33	1.27	4.2	3.96	3.65	2.9	2.95
*R*^2^	0.66	0.74	0.76	0.62	0.68	0.63	0.82	0.66	0.79

### Responses of nighttime NEE to soil temperature and soil moisture

**Figure 8:**
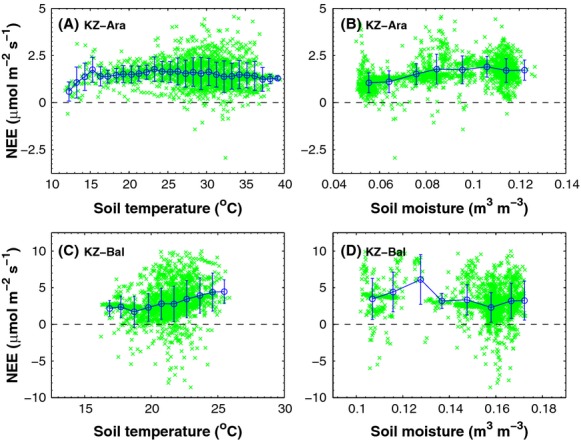
Response of half-hourly mean nighttime NEE to soil temperature and soil moisture during the study period (May–September) at the KZ-Ara (A and B) and KZ-Bal (C and D) sites. The blue open circle with line indicates bin-averages of 1.0°C and 0.01 m^3^ m^−3^ widths for soil temperature and moisture, respectively. Error bars refer to ±1 standard deviation.

The sensitivity of nighttime respiration to temperature was further inspected. Nighttime respiration is usually described as an exponential function (Q_10_ model) of near surface air or soil temperature (Xu and Baldocchi 2004; Reichstein et al. 2005). In most cases, Q_10_ model has been used separately at relatively short time periods, to avoid the confounding effects of phenology and soil moisture. At the KZ-Ara site, mean nighttime ecosystem respiration does not significantly respond to soil temperature but behave relatively constant at 1.5 *μ*mol m^−2^ s^−1^ in a wide range of soil temperature from 15 to 35°C (Fig. 8A). The variation of nighttime respiration tends to be large at high soil temperature, while mean nighttime respiration slightly increases with the increase in soil moisture (Fig. 8B). At the KZ-Bal site, the response of respiration to soil temperature exhibits an increasing trend, but the dependency between them is not statistically significant (Fig. 8C), while nighttime respiration does not significantly respond to the change in soil moisture (Fig. 8D).

Several attempts to fit Q_10_ models (Xu and Baldocchi 2004; Reichstein et al. 2005) showed that neither of these models was able to represent the response of nighttime NEE to variations of soil temperature for the two sites, even after categorized soil moisture into classes (results not shown). There is a relatively wide range of soil temperature at the KZ-Ara site, and independency of respiration on soil temperature may be explained by the small carbon pool in soil profile (Breckle et al. 2012). However, the response of nighttime respiration to soil temperature is clear as the potential of nighttime ecosystem respiration in grassland is larger at the KZ-Bal site. These results indicate the determinant of ecosystem respiration in different Central Asian ecosystems could be either soil carbon pool or environmental factors.

### Daily NEE dynamics during growing season

**Figure 9:**
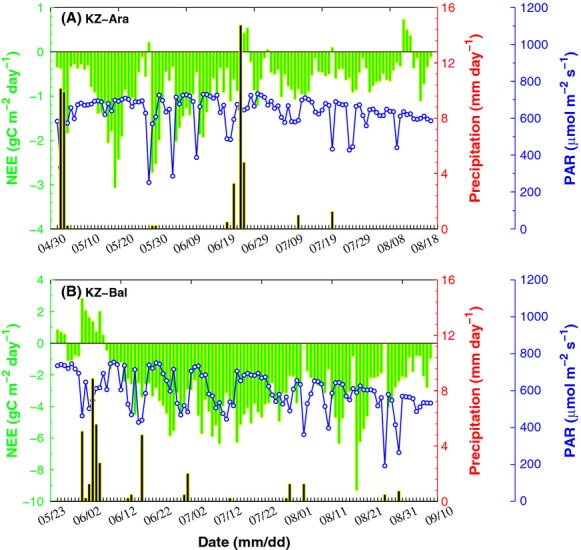
The daily NEE (green bar) accumulated by half-hourly values and daily precipitation (black bar with green outline) as well as mean daily PAR (blue line with open circle) during growing season at the KZ-Ara (A) and KZ-Bal (B) sites.

Figure 9 displays the dynamics of daily accumulated NEE as well as daily precipitation and mean daily PAR. Overall, carbon uptake rates of the Aralkum desert at the KZ-Ara site were lower than those at the KZ-Bal site. At both sites, daily NEE showed high variability during growing season, indicating that they are highly sensitive to the changes in environmental factors such as PAR and precipitation. The maximum daily NEE can reach up to −3 gC m^−2^ day^−1^ at the KZ-Ara site (Fig. 9A) and daily NEE can exceed −8 gC m^−2^ day^−1^ at the KZ-Bal site (Fig. 9B). Correspondingly, the magnitude of carbon loss on and after cloudy or rainy days at the KZ-Bal site was higher than that at the KZ-Ara site. Daily NEE at both sites can exhibit negative values, that is, net carbon uptake, on sunny days with high PAR (>600 *μ*mol m^−2^ s^−1^). On cloudy or rainy days, daily NEE at both sites tended to be positive, that is, net carbon release to the atmosphere. For instance, consecutive rainfall on the days 23–24 June caused noticeable carbon loss at the KZ-Ara site. Similarly, five consecutive days of rainfall from 30 May to 3 June led to consecutively significant and considerable carbon release (0.5–3.5 gC m^−2^ day^−1^) into the atmosphere at the KZ-Bal site. On other cloudy days, for example, the day on 27 May at the KZ-Ara site with low PAR of 250 *μ*mol m^−2^ s^−1^ showed a net carbon loss.

## Discussion

During the past decade, the net ecosystem CO_2_ exchange from desert areas has received much attention. Underlying reasons may come from the vast extent of arid and semi-arid ecosystems in terrestrial ecosystems (Dregne 1983), and the high variability of net ecosystem CO_2_ exchange, which is strongly dependent on climatic conditions, especially precipitation (Bell et al. 2012). Moreover, Wohlfahrt et al. (2008) reported that desert ecosystem in Mojave Desert of USA can act as a strong carbon sink whose capacity could be compared with many forests. Almost at the same time, a similar finding was reported in Gurbantunggut Desert in China, where the strong carbon sink was attributed to CO_2_ absorption by alkaline soil at nighttime (Stone 2008; Xie et al. 2009). Although lots of research can support the existence of carbonate dissolution (i.e., abiotic process) in alkaline soils (Serrano-Ortiz et al. 2010), the rate of carbon uptake reported by Xie et al. (2009) was questioned by Schlesinger et al. (2009).

The growing season net ecosystem CO_2_ exchange data that we have measured are the first data for desert ecosystem with alkaline soils in Kazakhstan which may offer, to certain extent, improved understanding on the carbon sequestration capacity of desert ecosystems in Central Asia. Although Liu et al. (2012a) have reported the annual net carbon balance based on daily integrated NEE data in the Gurbantunggut Desert of China, a similar desert ecosystem as the two sites in Kazakhstan used in the current research, the rates of daytime, nighttime, and diurnal variations of net ecosystem CO_2_ exchange were unknown. We addressed these questions and found that the diurnal course of the growing season net ecosystem CO_2_ exchange in the two desert ecosystems with highly alkaline soils followed clear sinusoidal pattern, which are quite similar as in crop, forest, grass ecosystems (Baldocchi and Meyers 1998; Falge et al. 2002), and the desert ecosystems in other areas where soil may not be alkaline (Bell et al. 2012). Net carbon release at nighttime and on cloudy and rainy days and net carbon uptake on daytime on sunny days are in consistent with ecosystems where biological factors dominated the variation of NEE. Then what is the effect of alkaline soil on net ecosystem CO_2_ exchange and what is the magnitude and aptitude of the contribution from abiotic processes in the desert ecosystems? Eddy covariance alone might be insufficient to answer this question. However, comparing the variations of NEE in the two sites with high alkaline soils in this research with that in other desert ecosystems and identifying the rates of NEE at daytime, nighttime, and its diurnal course could provide some insights on the mentioned questions.

Summarizing the previous reports on the net ecosystem CO_2_ exchange in desert ecosystems globally, the annual NEE has a very broad ranges of −127 to 258 gC m^−2^ year^−1^, although annual site-received precipitations were comparable (140–186 mm) (Table 2). Our measurements show a net ecosystem production that the growing season (May – September) of −86.6 and −297.8 gC m^−2^ at the KZ-Ara and KZ-Bal sites, respectively. Obviously, the estimates at the two sites did not take account of NEE out of the study periods which were mostly possible net carbon loss inferred from the monthly variations of NEE (Figs. 5 and 9). In addition, large carbon sink strength in the KZ-Bal site (−297.8 gC m^−2^) during the growing season was strongly related to the fact that the site was actually impacted by human interference (surrounding irrigated croplands and well-grown vegetation supplied by adequate soil water from adjacent acequia, see Fig. 1). The NEE in desert ecosystem is highly sensitive to environmental factors, especially precipitation (Bell et al. 2012; Liu et al. 2012a). The significant difference between the measured growing season NEE at the two sites also indicated that the EC-measured NEE can be strongly influenced by the specific location of the EC system established and the surrounding conditions (especially soil moisture and hence the vegetation condition), as addressed by Schlesinger et al. (2009).

**Table 2:** Comparison of annual net ecosystem CO_2_ exchange (NEE) using eddy covariance (EC) technique or equivalent experiments among different desert ecosystems in the world. Tair and Prcp represent mean annual air temperature and precipitation

Site	Longitude	Latitude	Soil	Dominant vegetation	Tair (°C)	Prcp (mm)	Annual NEE (gC m^−2^ year^−1^)	Period	Source
Aralkum Desert, Kazakhstan	61.08°E	45.96°N	Alkaline	Shrub	8.3	140	>−86.6	May–August, 2012	This study
Balkhash Lake, Kazakhstan	76.63°E	44.57°N	Alkaline	Grass	5.7	140	>−297.8	May–September 2012	This study
Gurbantunggut Desert, China	87.93°E	44.28°N	Alkaline	Shrub	6.6	150	−49 to −5	2006–2007	Liu et al. (2012a)
Gurbantunggut Desert, China	87.93°E	44.28°N	Alkaline	Shrub	6.6	150	−622 to −62	2005–2006	Xie et al. (2009)
Mojave Desert, USA	115.92°W	36.82°N	Loamy	Shrub	15.8	150	−110 to −102	2005–2006	Wohlfahrt et al. (2008)
Mojave Desert, USA	115.92°W	36.82°N	Loamy	Shrub	15.8	150	−127	2003–2004	Jasoni et al. (2005)
Baja California, Mexico	110.44°W	24.13°N	Yermosols	Shrub	23.8	174	−52 to −39	2002–2003	Hastings et al. (2005)
Baja California, Mexico	110.44°W	24.13°N	Yermosols	Shrub	23.8	174	−52 to 258	2002–2008	Bell et al. (2012)
Inner Mongolia, China	113.57°E	44.08°N	Loamy sand	Desert steppe	3.2	184	−7.2	2008	Yang et al. (2011)
Mongolia Plateau	118.89°E	41.79°N	Loamy sand	Desert steppe	6.7	180	43 to 48	2010–2011	Shao et al. (2013)
Tenger Desert, China	105.03°E	37.52°N	Sandy	Revegetation	10.6	186	−23.4 to −13.9	2009–2010	Gao et al. (2012)

**Figure 10:**
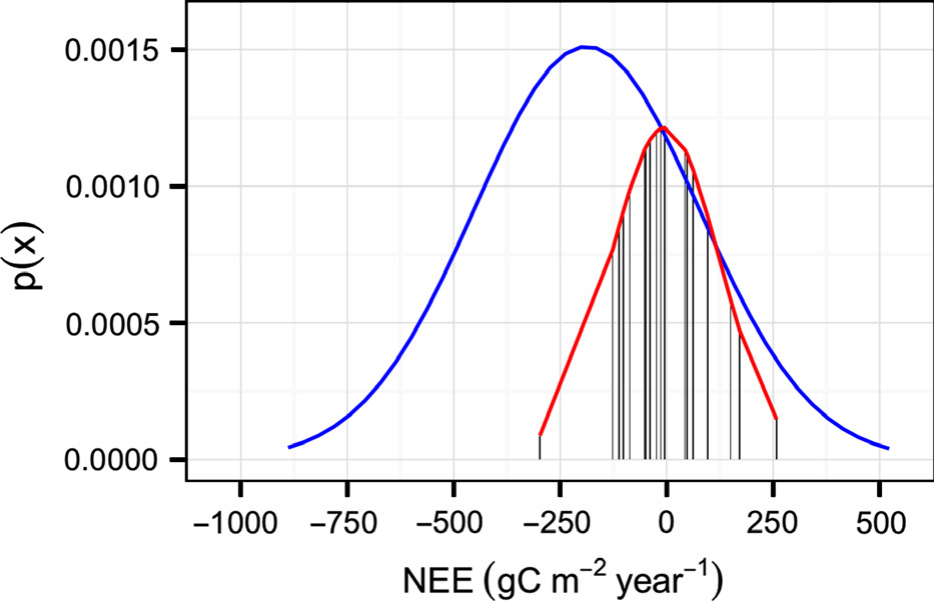
Gaussian probability distribution (p(x)) of published measurement of annual net ecosystem CO_2_ exchange (NEE) for global terrestrial ecosystems (blue line) and desert ecosystems (red line). For global data set, the mean is −183 and the standard deviation is 270 gC m^−2^ year^−1^ from 506 site-years of data (source: Baldocchi 2008). For desert ecosystems, data are retrieved from literature. In the superimposed Gaussian distribution for desert ecosystems, the mean NEE is −20 and the standard deviation is 190 gC m^−2^ year^−1^.

Global terrestrial ecosystem showed a quasi-Gaussian probability distribution with the mean NEE of −183 gC m^−2^ year^−1^ and the standard deviation of −270 gC m^−2^ year^−1^ based on 506 site-years of data (Baldocchi 2008). Using 18 site-years of measured NEE from desert ecosystems (Table 2), a superimposed Gaussian probability distribution showed the mean value with −20 and the standard deviation with 190 gC m^−2^ year^−1^. Desert ecosystems located in the right side of the global NEE distribution (Fig. 10), which indicated that the strength of NEE in desert ecosystems was lower than the global mean value. The NEE of desert ecosystems had a wide range from −250 to 250 gC m^−2^ year^−1^, and significantly influenced by annual precipitation (Bell et al. 2012) and human interference (for example surrounding acequia at the KZ-Bal site). The reported low annual NEE (<−100 gC m^−2^ year^−1^) was questionable (Schlesinger et al. 2009). The compiled 18 site-years of EC-measured annual NEE may exceed the −100 gC m^−2^ year^−1^ boundary (Fig. 10). However, desert ecosystems tend to be neutral or week sink of carbon in the long term.

Both sites of desert ecosystems with alkaline soils in Kazakhstan acted as obvious net carbon release at nighttime and on cloudy and rainy days, but net carbon uptake at daytime on sunny days (Figs 5 and 9). Comprehensively considering the strong dependency of daytime NEE on PAR, the responses of nighttime ecosystem respiration to soil temperature and moisture, and monthly variations of mean NEE, and their responses to precipitation (Figs. 9), we can conclude that biological processes of carbon cycle still dominated the net ecosystem CO_2_ exchange at the two desert ecosystems in Kazakhstan where soil was featured as alkaline and high soil pH value. Latest reports based on continuously measured soil respiration in alkaline soil exhibited significant net carbon loss at both daytime and nighttime (Ma et al. 2012). These recent findings were in contrast with the report (Xie et al. 2009) and hypothesis of Stone (2008), but consistent with the viewpoint of Schlesinger et al. (2009). Even recognizing the existence of CO_2_ absorption by alkaline soil (Serrano-Ortiz et al. 2010), the magnitude and aptitude of CO_2_ uptake by alkaline soils may not be noticeable and its contribution to net ecosystem CO_2_ exchange should be fairly limited.

## Conclusions

The present study provides the first insight on the diurnal, daytime, nighttime, and daily rates of net ecosystem CO_2_ exchange based on the measurements of EC in two sites of desert ecosystems with alkaline soils in Central Asia. The results found that the diurnal courses of NEE in each month followed clear sinusoidal patterns during growing season. Negative values of mean NEE were found at daytime on sunny days, indicating a net carbon uptake. In contrast, positive values of mean NEE were observed on cloudy or rainy days and at nighttime, which implied a net carbon source. Furthermore, strong dependency of NEE on PAR and the response of NEE to precipitation indicated that desert ecosystems with alkaline soils were still dominated by biotic factors, similar to other ecosystems, and abiotic CO_2_ absorption by alkaline soils may be trivial in terms of magnitude and aptitude.
